# How to Establish a Dental Practice

**Published:** 1897-10

**Authors:** N. S. Hoff


### How to Establish a Dental Practice.

BY N. S. HOFF, D. D. S.

The task of answering the question which stands as the subject of this paper has been assigned me by our worthy President, not because he had any reliable knowledge that I possessed the key to this important problem, as evidenced in any remarkable feat in this direction on my part, but I presume he solicited all the successful practitioners, including himself, and they have summarily declined to kill their golden goose. He very well knows that I am magnanimous to a fault, and am also pleased to see my literary effusions in print, and taking advantage of my weakness he has put me to a task which he thinks may floor me, or at least humble my vanity. I may satisfy his diabolical designs before I get through. But I have some ideas on this subject that I have never forced upon an indulgent audience, such as I have before me to-day, and I shall take heart and exploit them in my own way since I have your permission.

To begin with, there must be a personality. I am aware of the fact that many practices are established that are in certain directions and in some measure successful and which seem to be entirely impersonal. Patients visit the New York, or Boston, or Battle Creek dental parlors and put themselves under the care of no one in particular, but like chickens hatched in incubators, reared by a wooden box with cloth skirts and warmed by an oil lamp—a poor substitute for a vigilant, fat and well-fed mother-hen. But I do not claim to know anything about hatching out dental practices on the incubator plan. I wasn't brought up that way myself and of course I have little sympathy with this kind of practice building. In the kind of practice that I think we all crave, the personality, while not everything, is so important a factor that I give it the first place.

It is hardly necessary that I enumerate all the essential qualities which are considered necessary to the accomplishment of the greatest success. We can not regenerate men physically as we can morally. But I believe that where incurable physical deformities exist much can be done, with a proper appreciation of the requirements necessary for the adaptation of one to his environment. A man that is physically ill-formed may not be able to modify his conditions to make himself acceptable to everybody, and yet many an ill-natured person by persistent effort, thought and care-taking has been able to so modify his unattractiveness as to make himself entirely tolerable. I believe that all persons having such disabilities should be advised of their disadvantages before entering the profession. But when one is qualified and prepared for practice, these natural disabilities should not deter him from making a heroic effort to so conceal them with other attractive qualities as to have himself recognized in spite of his misfortune. Neither a blind man nor an armless man could practice dentistry successfully. There are many persons, however, trying to practice dentistry with defective eyes, and unconsciously perhaps, are not availing themselves of the aids a good oculist could give them. There are many persons with crippled fingers, stiff joints, and lack of acute tactile sense of the fingers, all of which stand in the way of successful practice. The skillful surgeon, a course of massage, or persistent attention to the care of the skin by systemic and local treatment would soon enable a bungling operator to appreciate the difference in the size of a lead pencil and nerve broach.

If it is important to take into account these hereditary and acquired physical disqualifications, and if it is possible to change them to more favorable conditions, how much more important is it that temporary disqualifications should be looked after carefully—conditions of neglect in clothing, toilet, or the presence of disease or its results. These may be and usually are offensive and will stand between dentist and patient to such an extent as to keep patient and dentist out of harmony with each other. No delicate and refined lady wants to have for her dentist a man who smokes bad cigars, pipe or worse, cigarettes, neither will such a one long tolerate a person whose breath suggests the garbage pail, from his indulgence in malt liquors, fragrant cheese and odoriferous relishes. In this climate catarrhal troubles are common, and without proper care the breath under such circumstances is bound to be offensive. If perchance, as frequently happens, there is also a complication of indigestion, the conditions are certainly adverse and demand peremptory correction. Eruptions on exposed parts of the body and other offensive conditions are to be scrupulously avoided. Strict attention to personal habits of cleanliness, combined with clean and well-selected clothing and ornaments, are of the greatest moment, and scrupulous cleanliness both internally and externally are necessary and to be constantly striven for and kept in mind. It is above all necessary that the dentist should be above reproach or suspicion morally. It is not for me to formulate or quote a moral code for any one. The dentist should be an honorable gentleman, and while it is not necessary that he be religious, it will not be any drawback to be known and recognized as an active and energetic member in good standing of some religious organization. Of course I would not have any one infer that a church-membership is to be valued as a business aid only. A person who would make use of a religious or any other sacred organization for his own selfish purposes deserves the severest contempt of every right-minded person. But I hold that it is legitimate for a person to make use of the church and any or all other organizations which he can command to exalt and ennoble his own character. And no personality which is not in some way influenced by Christian religion or morals is fully equipped to make the most out of his opportunity to serve a large and appreciative clientele.

Again, an educated person will have decided advantage over one whose entire store of knowledge is confined to his profession, or is limited and characterless. Patients respect and want culture in their dentist as they expect and seek it in their preacher, doctor or lawyer. The broader a man's knowledge the wider will be his influence professionally. What are the essentials? Every dentist who hopes to get and retain the confidence and support of his patients must be well qualified to perform any and all ordinary service in a good and substantial manner. This implies a thorough course of training in all the technical functions of dentistry as well as such theoretical study as shall be necessary to qualify him to correctly diagnose diseased conditions and to apply proper and adaquate remedies for relief. To obtain this degree of proficiency he must set a high mark for attainment; one that can not be reached by a three years' or five years' course of study in any dental educational institution, but which shall only be reached after years of careful and painstaking effort to do the best possible for every case undertaken; simple as well as difficult cases should be subjected to the closest scrutiny that nothing of value or interest in any case may be overlooked. The early establishment in one's mind of a systematic method in making diagnoses, and the treatment of every case by principles rather than without interest or mechanically, is of the utmost importance. No dentist who performs his work in a perfunctory manner will respect or love his work or calling, and his patients will know of it before he fully appreciates it himself. The man who cobbles a shoe takes pride in his work in proportion to the skill and thought he puts into it. The scientist, diplomat or statesman takes pride in his work only so far as he has put his best into it, and the dentist who will put all the brains he can into his work will soon find his heart enlisted, and it will be marvelous indeed to have such a combination defeated. We always find that the man who gets thus interested in his own work and becomes attached to his calling, and finds himself appreciated by the community in which he lives, begins to think of his professional brethren and he wants to know what they are thinking and how they get along with their work, and he subscribes for the dental magazines and begins to read, and then he wants to see these men who write and talk in society meetings; he wants to ask them some questions that he finds difficult to adjust, and so he joins his local, state or national dental society, and he attends the meetings as religiously as he does his church prayer-meeting, and he feels in duty bound to take his part in the dental meeting just as much as in his church prayer-meeting, and thus he gets interested in his profession, and his patients know it without any announcement on his part through the press and are glad of it and proud of their dentist, not because he announces in all the local papers that he is such a big man away from home—that he has been summoned by the dentists in national assemblage to tell them how he does it—but because they are glad that he is broad and liberal enough to take an interest in his calling and is willing to sacrifice his time and money to get on in the calling to which he is devoting his life.

A very wealthy and intelligent man, a warm, personal friend of mine, said to me, when I was a young professional man, that he wanted to say that he would like to come to me professionally, and also send his family to me, but he said he had always gone to another dentist, and had paid him large fees willingly, because he knew that he gave him his best services, and further that he believed he was a thoroughly professional man, and would use whatever money he paid him for the advancement of his profession. To me this statement was an eye-opener, and I have many times wondered why all people did not not hold the same view. I really believe more of our patients have something of this idea than we think, though they may not be conscious of it or be able to formulate it into any well defined-theory for action.

The point I want to make is that if we expect to attract intelligent and discriminating people professionally, we must be the embodyment of the highest prevailing or known ideas of the professional; we must actually live the life, not theoretically nor ostentatiously, but honestly and conscientiously.

There is a third qualification that it seems to me is about as important as the two to which I have called attention: that is the faculty or ability to conduct professional business on legitimate and conscientious business principles. The comment is frequently made that professional people are notably deficient in business methods or habits. Few professional men accumulate large properties, although they may do considerable business and at good profit. There are various reasons for this, all of which I will not take your time to rehearse; but it is true that a professional man with a large practice has no time to cultivate business habits if he is inclined to do so or even determined to do so, and consequently he must entrust these matters to others. If he be fortunate enough to employ the right person all goes well, but frequently he will see the accumulation of years vanish because his money has been unfortunately or unwisely invested. It is said that ninety percent of business men fail, and the professional man who places his confidence in a class of men who default in so large a percentage must not be surprised if they do not always trust wisely and well. There is no question but that professional men ought to lay by a portion of their earnings and begin early to do so, and keep everlastingly at it, and employ the safest means available for caring for these accumulations until such time as they are of sufficient amount to purchase productive real estate or safe mortgages on good real property.

But there is another way in which dentists, doctors and lawyers are frequently accused of being bad business men. This is in not meeting their obligations promptly. There are of course rascals in the dental as well as other callings; but all dentists who do not pay their debts promptly are not rascals, at least not intentionally. Many professional men are tempted or educated into this lax habit of not paying bills promptly by their patients. Doctors are probably the worst paid of all professional men, perhaps lawyers next, and, owing to their close relationship, dentist's bills are put off until the landlord, the grocer and baker are all paid, and if anything is left the dentist gets it, and after he is paid the doctor may get a portion. A dentist in full practice realizes that he is making a certain amount of profit, and naturally thinks he has the right to use it for such necessities and luxuries as he may desire, and consequently spends or contracts to spend much of his money before it is collected. Or if he is not so reckless, he reasons that when others owe him, he has a right to hold off payment of his debts until some convenient time. This is a kind of logical justice that has no support in the moral code. Nevertheless, it operates largely, and with men who would not like to be called selfish or dishonorable. The moral is, that, to have the confidence of the business community, a dentist should pay his debts as promptly as it is possible, and never to spend his earnings until in hand. To do this he will need to give some thought to the manner of making his collections regularly and systematically, and without offense to his patrons. This brings up the whole subject of keeping accounts and records, and the best methods for regulating fees and of making collections which I have no time to discuss here; but I do want to say that the business part of conducting a dental practice is exceedingly important, and should not be carelessly nor thoughtlessly managed. I have heard it said of dentists that their fees are often based not on the character and amount of the service rendered so much as on the ability of the patient to pay or the pressing needs of the dentist. I once had a dentist relate to me how he worked the fee he first planned to charge, to more than double, simply because he had a willing patron and his wife insisted on his buying a handsome velvet cloak for their daugher. Such business ideas are not only shiftless, but contemptible and dishonorable, and no man who practices them will permanently succeed, nor should he. Such habits will unquestionably demoralize one's business capacities and wreck his professional life, because the aim will eventually be not how well can the work be done, but how quickly and at the least possible outlay of effort, mental and physical. Its result is to be found in the miserable attempts we see all around us, to get the attention of the unwary by resorting to all sorts of schemes to get the public to take notice of the great and only Dr. Quack, who extracts teeth free, without pain, from 9.00 to 12.00 o'clock on Saturdays, etc., etc. Does any intelligent, honest or well-bred and self-respecting man want to have such a name or notoriety? So any one seeking such notoriety a more commendable method, and one not likely to injure innocent and unsuspecting persons, is to get together a menageriec of albino children, bearded women, double-headed calves, big snakes, etc., and erect a tent on some vacant lot, and with a hand-organ to attract the crowd, he can gratify his ambition of being seen and heard.

The vulgar display of one's profession is not to be commended in any sense. It is not a good business idea for a young man, when he decides upon a location for establishing a practice, to embarrass his future business success with an extravagant outlay for furniture, equipment or excessive office rent, which he has no immediate nor remote probability of being able to pay. I

would not, on the other hand, advise a man to fit out so meanly that refined persons would hesitate to approach his premises, or that he would be handicapped in his work by inferior or inadequate accessories. What I wish to say is that no man is justified in making what exceeds the bounds of good judgment in assuming financial obligations which may cause him to lose his professional and business integrity. The chances of recovering ground once lost in this way are few in comparison, and it is better far that time should be sacrificed than honor or professional standing in the community and among the professional brethren.

My conclusion, then, in regard to qualification is that one should continually study to make himself personally agreeable to those whom he would serve; he should strive by professional reading, study, work, association and every other legitimate way to adapt himself to his career, and at the outset establish correct and conservative business methods and practices.

A preparation of this kind will fit one to select a location with reasonable hope that he, in time, will certainly attain a considerable measure of success.

The second and perhaps equally-important phase of this question to which I wish to call your attention is that of a location. I once asked a well-known man, who manages one of the largest dental schools in this country, what advice he gave to his students when they asked him where they should locate. He said he was in the habit of telling them to select a community where there were prosperous people. This answer it seems to me has some elements of worth in it, but there are practical difficulties which make it impossible for every one to avail himself of it. There are not prosperous communities enough to go around, and besides these prosperous communities are generally well supplied. This suggestion is filling our cities with dentists, and creating conditions, the result of competition which do not make for the upbuilding of high professional character nor friendly professional intercourse. The graduate just out of college thinks he is fully qualified to cater to the dental needs of the most intelligent and wealthiest people in the city, and he imagines that all he needs to do is to rent an office in a high-toned building on the best street, just across the way from the leading dentist of the city, and equip it with velvet carpets, mahogany furniture, electric machines, etc., and the nabobs of the city will fall over themselves getting to him for his services, and that wealth will soon be his "to burn."

The college graduate of to-day knows more than he did twenty years ago, and he thinks he knows more and can do more satisfactory dental work than the graduate of thirty or forty years ago, and it sometimes happens that he does and can, but too often there is a long season of quiet and waiting that is powerfully weakening and trying to one's nervous and mental equilibrium. Happy and fortunate is the one who is able to stand the long wait, and finally begins to experience the cheer of having occasional appreciative patients. With many, this period of waiting is so monotonous that they can not endure it, and forgeting the good precepts of their teachers in college, they use the idle time conjuring up some scheme of inducing the unwilling to come to them for professional services.

The character of these efforts are familiar and nothing will be gained by a rehearsal of them here; suffice it to say that they usually end in bringing reproach and shame upon an honored profession and professional ostracism to him who indulges. What is the trouble and where is the remedy? Are there too many dentists?

The trouble is caused by the all-American temptation to be rich or famous quickly and for the young man to think that he can step out of his father's home, where he has been accustomed to comfort and luxury, may be, and that he must start in life from his father's position, which represents the work of many years of self-denial, and jump speedily to better circumstances. The new dentist in town thinks he can, by starting in with new furniture and equipment and the latest ideas of progressive teachers, make at one bound all that has taken years of toil and labor for his older professional brother to accumulate. He thinks he can do as good or better work than the old man with failing eyesight, but he always fails to take into account the safe judgment which has come to be the consolation of and which has established the most enduring, confidential relations between the old dentist and his patients. These associations of years can not and should not be so easily disturbed. The trouble is the new dentist has made a mistake in thinking there are those to serve in the location he has selected. He finds them already well and satisfactorily cared for, and the only way he can move them to change is by some unfair inducement. The cities as a rule are well supplied with dentists. The rich and well-to-do are particularly well provided for, but there is a large class of people in every city, which, could it receive competent dental service, would not only appreciate it but be willing to make some remunerative exchange for it. I refer to those districts in almost every city where live the mechanical and laboring classes, or people who receive small wages and support large families. These people are honorable and well-meaning and have some ideas of hygienic living and so far as they are able they know the value of and do what they can to take care of their teeth. They can not patronize a dentist whose time is worth from $5 to $10 per hour, and they are compelled to go to the advertising man, who makes no suggestion to them looking to the saving of their teeth when he finds they can not pay a good fee, but recommends extracting and cheap rubber plates as the easiest way of getting what little he can out of them. I have for a long time been of the opinion that there are men practicing dentistry on Woodward Avenue in Detroit, who should be doing their duty out in the various factory districts, where there are people who need dentists as badly as the Woodward Avenue dentists need patients. It may be said that no dentist could afford to do this kind of missionary work, perhaps not all his life, but it seems to me any young college man who had the proper spirit, that would enable him to locate in the midst of these people and identify himself with them and their interests, could in a few years have more experience, and consequently better judgment than he would be able to obtain by many years of waiting and worrying in some more favored locality. Suppose he could not get five dollars per hour for his services, he could perhaps get one dollar per hour and could work ten or fifteen hours per day after he had gained the confidence of the people, and in place of paying $50

per month for an office, he could afford a comfortable house where he could begin a home for two or three months' rent of a down-town office, and by careful work and saving for a few years he would be sufficiently free-handed to move into desirable quarters in a more desirable part of the city, taking with him his more desirable patients, and there would be an opening left for some other young man to occupy for his own as well as the good of the community. The establishment of good dental offices in these outlying districts, it seems to me, would be one of the most christian and benevolent schemes of the time, and would do more to lessen the influence and demand for the cheap down-town dentists than any other thing.

Of course, there are circumstances which should change the plan of beginning a practice. If a man graduating from a school has favorable opportunities for establishing himself this plan should be modified. If he is thoroughly qualified and has money to sustain him until such time as he shall have sufficient income from his practice to sustain himself, or because he has unusual opportunities to be assisted into practice he probably would not gain anything by locating in a portion of the city where he did not intend to remain and where he would have to give his services for very small fees.

What I have said as to location in regard to these factory districts will apply to small country towns or villages. There are many small places in the country which are without the facilities for having dental services done at home, and there are enough people in these small places to support a man of small requirements, or will be after they have been educated to become good patients. Sometimes two small towns close together can be served by an energetic man to his own and the people's welfare.

If it is not practicable for one to establish himself in either of the ways I have mentioned above, there are two other ways of obtaining a practice that it seems to me are worthy of consideration and have decided advantages, one is to secure a position as assistant to some established dentist with the idea of eventually by purchase secure an interest in a business already established.

This plan has many advantages. It gives a young man a chance to learn from one of experience how to handle patients and conduct business. It also gives him a chance to perfect his judgment under the advice and with the counsel of an experienced practitioner. This method is probably, all things considered, one of the best methods of getting into practice in a legitimate and satisfactory way. The other method is to purchase outright the practice of another practitioner. This, I maintain, is a business way of securing dental patronage and is legitimate, and where the parties to the sale are honorable I am confident that the sale of a dental practice is desirable and can be profitably and successfully accomplished. The price one pays for a practice that can be transferred in this way will soon repay the purchase price and save years of tedious waiting. The question of where to locate, whether town or city, will of course need to be decided by each individual to suit his own tastes or inclinations and his desire to live in the city or country.

The irritation caused by the crowding of new men into towns, cities and localities where there is no apparent field for them, and where they will if they get any business at all be compelled to secure it at the expense of some one who has none to spare, can, it seems to me, be avoided by some concerted effort on the part of the profession to give advice to new men entering the profession and in a spirit of liberality. Some such concerted action on the part of the profession will help to satisfy the profession more and more until we have a harmonious profession with one purpose and aim, that of giving to the people the adequate service they require.

